# Risk Factors for Long-Term Mortality after Hospitalization for Community-Acquired Pneumonia: A 5-Year Prospective Follow-Up Study

**DOI:** 10.1371/journal.pone.0148741

**Published:** 2016-02-05

**Authors:** Jan C. Holter, Thor Ueland, Pål A. Jenum, Fredrik Müller, Cathrine Brunborg, Stig S. Frøland, Pål Aukrust, Einar Husebye, Lars Heggelund

**Affiliations:** 1 Department of Internal Medicine, Drammen Hospital, Vestre Viken Health Trust, Drammen, Norway; 2 Research Institute of Internal Medicine, Oslo University Hospital Rikshospitalet, Oslo, Norway; 3 Institute of Clinical Medicine, Faculty of Medicine, University of Oslo, Oslo, Norway; 4 K.G. Jebsen Inflammatory Research Center, University of Oslo, Oslo, Norway; 5 Department of Medical Microbiology, Drammen Hospital, Vestre Viken Health Trust, Drammen, Norway; 6 Department of Microbiology, Oslo University Hospital Rikshospitalet, Oslo, Norway; 7 Oslo Center of Biostatistics and Epidemiology, Research Support Services, Oslo University Hospital, Oslo, Norway; 8 Section of Clinical Immunology and Infectious Diseases, Oslo University Hospital Rikshospitalet, Oslo, Norway; University of Louisville, UNITED STATES

## Abstract

**Background:**

Contributors to long-term mortality in patients with community-acquired pneumonia (CAP) remain unclear, with little attention paid to pneumonia etiology. We examined long-term survival, causes of death, and risk factors for long-term mortality in adult patients who had been hospitalized for CAP, with emphasis on demographic, clinical, laboratory, and microbiological characteristics.

**Methods:**

Two hundred and sixty-seven consecutive patients admitted in 2008–2011 to a general hospital with CAP were prospectively recruited and followed up. Patients who died during hospital stay were excluded. Demographic, clinical, and laboratory data were collected within 48 hours of admission. Extensive microbiological work-up was performed to establish the etiology of CAP in 63% of patients. Mortality data were obtained from the Norwegian Cause of Death Registry. Cox regression models were used to identify independent risk factors for all-cause mortality.

**Results:**

Of 259 hospital survivors of CAP (median age 66 years), 79 (30.5%) died over a median of 1,804 days (range 1–2,520 days). Cumulative 5-year survival rate was 72.9% (95% CI 67.4–78.4%). Standardized mortality ratio was 2.90 for men and 2.05 for women. The main causes of death were chronic obstructive pulmonary disease (COPD), vascular diseases, and malignancy. Independent risk factors for death were the following (hazard ratio, 95% CI): age (1.83 per decade, 1.47–2.28), cardiovascular disease (2.63, 1.61–4.32), COPD (2.09, 1.27–3.45), immunocompromization (1.98, 1.17–3.37), and low serum albumin level at admission (0.75 per 5g/L higher, 0.58–0.96), whereas active smoking was protective (0.32, 0.14–0.74); active smokers were younger than non-smokers (*P* < 0.001). Microbial etiology did not predict mortality.

**Conclusions:**

Results largely confirm substantial comorbidity-related 5-year mortality after hospitalization for CAP and the impact of several well-known risk factors for death, and extend previous findings on the prognostic value of serum albumin level at hospital admission. Pneumonia etiology had no prognostic value, but this remains to be substantiated by further studies using extensive diagnostic microbiological methods in the identification of causative agents of CAP.

## Introduction

Community-acquired pneumonia (CAP) is a major cause of morbidity and mortality worldwide [1]. Besides remarkable short-term (30-day) mortality rates consistently ranging from 10 to 12% [1, 2], CAP is also associated with substantial long-term mortality after recovery from the acute episode [3, 4], with 5-year mortality rates exceeding 50% [2, 5]. In addition, CAP itself confers an independent risk of long-term mortality [5–9], but it is not clear what factors explain this excess mortality risk observed in patients with CAP.

Respiratory and cardiovascular diseases (CVD) and malignancy are the most common causes of death among patients with CAP [6, 7, 9–14], suggesting that they are exposed to a wide range of risk factors. Probably the most consistently identified risk factors encompasses older age, severity of illness [2, 7, 15–19], and existing or new-onset comorbidities, including neurodegenerative disorders, CVD, chronic obstructive pulmonary disease (COPD), diabetes, and malignancy [3, 4, 10, 13]. In addition, acute kidney injury and acute cardiovascular events, both during hospitalization and after discharge, may occur in patients with CAP, both of which have a strong impact on patients’ outcomes [20–23], but the mechanisms remain poorly understood (e.g., persistent systemic inflammatory activity, pro-thrombotic state) [14, 19, 21]. Moreover, apart from host and host-derived factors, it has been shown that pneumococcal pneumonia is associated with mortality far beyond the acute infection [24].

Understanding host as well as microbial causes of CAP that contribute to long-term impact is important to ensure good patient outcomes [25]. Although the latter study suggests that identifying the causative agent may have a long-term prognostic value in patients with CAP, most previous studies have used CAP as a generic disease without regard for specific microbial etiology, or have been hampered by low yield of microbiological assessments before the introduction of molecular diagnostics [6, 11, 13, 24, 26–28]. Importantly, CAP can be caused by a number of different pathogens, either alone or as part of a polymicrobial infection, including some that are characterized by a severe course (e.g., *Streptococcus pneumoniae*, influenza viruses) and some that are less severe, or difficult to detect [25]. Indeed, we have recently reported that a high microbial yield was achieved in a prospective cohort of adult patients with CAP by combining conventional (bacterial cultures, urinary antigen assays, serology) and molecular microbiological methods [29]. Here, we examined survival, causes of death, and risk factors for all-cause mortality over a longer period of follow-up in the patients who survived their initial CAP hospitalization, with emphasis on demographic, clinical, laboratory, and microbiological characteristics.

## Methods

### Study population and design

This cohort study was carried out in an acute care 270-bed general hospital in Drammen, Vestre Viken Health Trust, serving a source population of 160 000 in South-Eastern Norway. All adult patients (aged ≥18 years) with suspected pneumonia who were admitted to the Medical Department were prospectively recruited between January 1st 2008 and January 31st 2011. Patients were screened within the first 48 hours of admission for study inclusion by determining whether or not they met the criteria for CAP as defined by (i) presence of a new pulmonary infiltrate on chest radiograph, (ii) rectal temperature >38.0°C, and (iii) at least 1 of the following symptoms or signs: cough (productive or non-productive), dyspnea, respiratory chest pain, crackles or reduced respiratory sounds. Patients were excluded from the study when the chest radiographic examination showed non-infectious causes such as pulmonary infarction, tumor or bronchiectasis, or if the patient had been hospitalized within the past 2 weeks. Immunocompromized patients (i.e., primary or acquired immunodeficiency, active malignancy, patients using immunosuppressive drugs) were not excluded from the study to reflect the total population being referred to this local hospital. The inclusion process for the study population (n = 267) has been described elsewhere [29] and is summarized in S1 Text. Patients who died during hospitalization were excluded (n = 8), i.e., only patients surviving their initial hospitalization for CAP were included in the present analysis (n = 259).

Patients were followed from the date of hospital discharge until the closing date of December 31st 2014. Patients who died were considered responders at their death dates and those who survived after the closing date were considered censored. Patients lost to follow-up were censored at the time of last known contact. All patients provided written informed consent. The study was approved by the Regional Committee for Medical and Health Research Ethics in South-Eastern Norway (reference number: S-06266a) and a waiver of consent was obtained from the committee to link patient data to death certificates (2012/467 A).

### Demographic, clinical and laboratory data collection

Data on age, sex, smoking status, nursing home residency, duration of symptoms before admission, preexisting comorbidities, influenza and pneumococcal vaccination status, radiographic findings, and laboratory values were collected within 48 hours of admission. Chest radiographic patterns were examined by an independent experienced radiologist and categorized as unilateral or bilateral lung involvement. Severity of illness was measured by intensive care unit (ICU) admission during hospitalization, and by the validated CURB-65 scoring system [30], composed of the variables (accounting for one point each): confusion of new onset, urea >7 mmol/L, respiratory rate ≥30 breaths/min, blood pressure (systolic <90 mmHg or diastolic ≤60mmHg), and age ≥65 years, obtained by abstraction of the most abnormal values documented in medical records within the first 48 hours of admission. This prediction rule enables patients with CAP to be stratified according to increasing risk of 30-day mortality: <3% mortality for scores of 0–1, 9% for score of 2, and 15–40% for scores of 3–5. In the present study, patients with a CURB-65 score of <3 were classified into low-risk, and ≥3 into high-risk groups.

### Definition of variables

*Nursing home* was defined as patients who resided in a nursing home or a long-term care facility. *Active smoking* was defined as self-reported smoking. *COPD* was defined as a physician’s diagnosis in the medical record, or prior history of tobacco smoking and at least 2 of the following: dyspnea, chronic cough or sputum production. *CVD* included the occurrence of (i) coronary heart disease, defined as evidence of 1 or more of the following: myocardial infarction, coronary artery bypass graft surgery, percutaneous coronary revascularization, or treatment for angina pectoris; (ii) heart failure, defined as a physician’s diagnosis in the medical record, or evidence of at least 2 of the following: heart failure symptoms at rest or during activity, objective evidence of cardiac dysfunction at rest by echocardiography, or clinical response to treatment directed at heart failure; (iii) cerebrovascular disease, defined as clinical diagnosis of stroke or transient ischemic attack, or stroke documented by magnetic resonance imaging or computed tomography; (iv) peripheral artery disease, defined as intermittent claudication or prior bypass for arterial insufficiency, gangrene or acute arterial insufficiency; or (v) untreated thoracic or abdominal aneurysm (≥6 cm). *Diabetes mellitus* was defined as a physician’s diagnosis in the medical record, or evidence for 2 fasting serum glucose measurements >7 mmol/L or 2-hour post-75 g oral glucose tolerance test serum value ≥11.1 mmol/L, or a casual serum glucose ≥ 11.1 mmol/L. *Renal disease* was defined as a history of chronic renal disease or abnormal blood urea nitrogen and creatinine concentrations documented in the medical record. *Liver disease* was defined as clinical or histologic diagnosis of cirrhosis or another form of chronic liver disease, such as chronic active hepatitis–except for autoimmune hepatitis (outlined below). *Dementia* was defined as documentation of chronic cognitive deficit based on disease history. *Neurological disease* included the occurrence of (i) central nervous disease, defined as any seizure disorder (i.e., epilepsy, convulsions); or (ii) neuromuscular disease, defined as Parkinson’s disease, amyotrophic lateral sclerosis, multiple sclerosis, myasthenia gravis, Charcot-Marie-Tooth disease, and/or dense hemi- or paraplegia. *Immunocompromized host* included the occurrence of (i) primary or acquired immunodeficiency, defined as antibody deficiency, HIV, organ transplant, and/or receiving chemotherapy and/or radiation therapy within the past 3 months; (ii) active malignancy, defined as any cancer except basal–or squamous–cell cancer of the skin that was active at the time of presentation or diagnosed within 1 year of presentation; or (iii) immunosuppressive drug use, defined as any use of systemic steroids, Azathioprine, TNF-alpha inhibitor, Cyclosporine, Cyclophosphamide or Methotrexate within the past 3 months. *Autoimmune disease* was defined as rheumatoid arthritis, systemic lupus erythematosus, inflammatory bowel disease, autoimmune hepatitis, Sjogren’s disease, and/or psoriasis.

### Microbiological specimen collection and methods

The specimen collection procedure and the microbiological methods used have been described previously [29]. In brief, for all 267 patients enrolled in the study attempts were made to collect two paired blood culture samples (n = 267), a sputum sample (n = 165) and a nasopharyngeal swab sample (n = 263) for bacterial culture, and a urine sample (n = 262) for *S*. *pneumoniae* and *Legionella pneumophila* serogroup 1 antigen testing. In addition, samples were obtained from the nasopharynx (n = 262) and the oropharynx (n = 262) using flocked swabs (Copan Flocked Swabs and UTM-RT Transport Medium System, Brescia, Italy) for analysis by real-time quantitative PCR for *S*. *pneumoniae*, and by real-time PCR assays for *Mycoplasma pneumoniae*, *Chlamydophila pneumoniae*, *Bordetella pertussis* and 12 types of respiratory viruses: adenovirus, influenza A and B viruses, H1N1, parainfluenza viruses types 1–3, metapneumovirus, rhinovirus, enterovirus and respiratory syncytial virus (A and B). Paired serum samples during the acute (n = 250) and convalescent (n = 229) phases of infection (separated by approximately 6 weeks) were also obtained for serological detection of *M*. *pneumoniae*, *C*. *pneumoniae*, *B*. *pertussis*, and influenza A and B viruses. Bronchoalveolar lavage (n = 8) and diagnostic thoracocentesis (n = 14) were performed on medical indication. Sputum samples and bronchoalveolar lavage were examined, if medically indicated, by use of real-time PCR detection of *L*. *pneumophila* and/or *Pneumocystis jirovecii*.

Of the 259 patients discharged alive (i.e., analysis cohort), 63% had an etiologic CAP diagnosis established. Based on the etiology, patients were categorized into 4 groups: (i) pure bacterial, (ii) pure viral, (iii) viral–bacterial, and (iv) no etiology established. In addition, patients with pneumococcal etiology (i.e., both bacteraemic and non-bacteraemic cases), influenza virus infection, and bacteraemia *per se* were studied separately.

### Assessment of mortality and causes of death

Deaths during the follow-up period were determined utilizing data from the Cause of Death Registry kept by the Norwegian Institute of Public Health. We obtained death registry information from January 1st 2008 to December 31st 2014. Each Norwegian inhabitant is registered with a unique lifetime personal health number. All deaths, including date of death, are reported by doctors who are required to complete a death certificate, and death certificates are routinely collected by the registry for coding of information according to the International Classification of Diseases 10th revision (ICD-10) coding system. The coverage of this register in Norway is 100%, thus mortality could also be ascertained for persons who left the region. In order to assign cause of death (i.e., the underlying cause of death), the registry routinely processes the ICD-10 codes from the death certificates using the international Automatic Classification of Medical Entry (ACME) system [31]. This system facilitates comparison of causes of death between countries. We used the output diagnoses from ACME reported in ICD-10 codes and categorized them into 5 groups according to organ system for analysis as described by Bruns et al. [7]: (i) pneumonia, (ii) COPD, (iii) vascular disease, (iv) malignancy, and (v) others.

### Statistical analysis

Categorical variables were expressed as counts and percentages. Continuous variables were presented as mean and standard deviation (SD) for normally distributed data, or median (25th–75th percentiles) for skewed data, as evaluated by the Kolmogorov-Smirnov test.

Survival from the time of hospital discharge until emigration, death or end of the follow-up period was described for all survivors of the initial hospitalization using Kaplan-Meier plot.

In a predictive strategy (i.e., no priority given to a specific hypothesis), risk factors for long-term mortality after hospitalization for CAP were assessed by Cox proportional hazards models using the purposeful selection of variables method described in Hosmer et al. [32]. With a mortality outcome of 30.5% (n = 259), we had 80% power to detect a hazard ratio (HR) of at least 1.43 using a significance level of 5%, and the Cox regression model could include 8 independent variables according to the rule of 1 covariate per 10 events. The variables for our model were decided *a priori* based on known and potential risk factors. The purposeful selection method first evaluates univariable associations and selects variables with the predetermined arbitrary significance level as candidates for the multivariable analysis. We chose a *P* cut-off point of < .15 because larger values would increase the risk of selecting less important variables at this initial stage. Additionally, variables with more than 15% missing values were not considered [33]. Variables were kept in the multivariable model if significant (*P* < .05) or if the variable was identified as a confounder (percent change in any remaining parameter estimate greater than 20% as compared with the full model). The partial likelihood ratio test was performed to compare models when removing covariates. Next, any variable not selected for the original multivariable model was added back one at a time, with significant covariates and confounders retained earlier (*P* < .10). Competing models were compared by using both the Akaike and Bayesian information criteria (AIC/BIC). This model-fitting process ended when no additional variables entered the model. The effect was quantified by HRs with 95% confidence intervals (CIs). Multivariable analyses were preceded by estimation of correlation between risk factors. The proportional hazards assumption was evaluated using Shoenfeld’s test and residual plots for each individual independent variable in the model and by plotting the logarithm of the integrated hazards (log-log survival plots), and these assumptions were met. Evaluation of the predictive accuracy of the model was assessed by calibration and discrimination. Calibration was evaluated by plotting Cox-Snell residuals and discrimination by Harrell’s c index (*c*-index). If the *c*-index is >0.7, it can be concluded that the model has an acceptable discriminatory capability.

The observed mortality in our cohort of CAP patients was compared to the expected total mortality of the Norwegian population stratified on age and gender. This permitted estimation of standardized mortality ratios (SMRs) with 95% CIs. The method consists of comparing age and gender specific rates of death (expected deaths, E) of a standard population, the total Norwegian population, to the observed deaths (O) in the population of interest, the present cohort. Tables, available online [34], giving the incidents of deaths per 100 000 inhabitants per year among women and men in different age groups were used. SMR was estimated as SMR = ∑O*i* / E*i* at age and gender strata [35]. The 95% CI was estimated as SMR ± 1.96 × SE(SMR), where SE(SMR)=SMR/√∑Oi, E*i* the summation of expected deaths, and O*i* the summation of the observed deaths. SMR above 1.00 means that a higher death rate is observed in the cohort than expected in the total national Norwegian population. If SMR is less than 1.00, fewer deaths are observed than expected. The SMR method of adjustment merely controls for gender, age and geographical area.

Data were analyzed using IBM SPSS Statistics 20.0 (IBM SPSS, Chicago, IL) and STATA 13.0 (STATA, Chicago, Ill., USA) software; all tests were two-tailed.

## Results

### Survival

**Fig 1:**
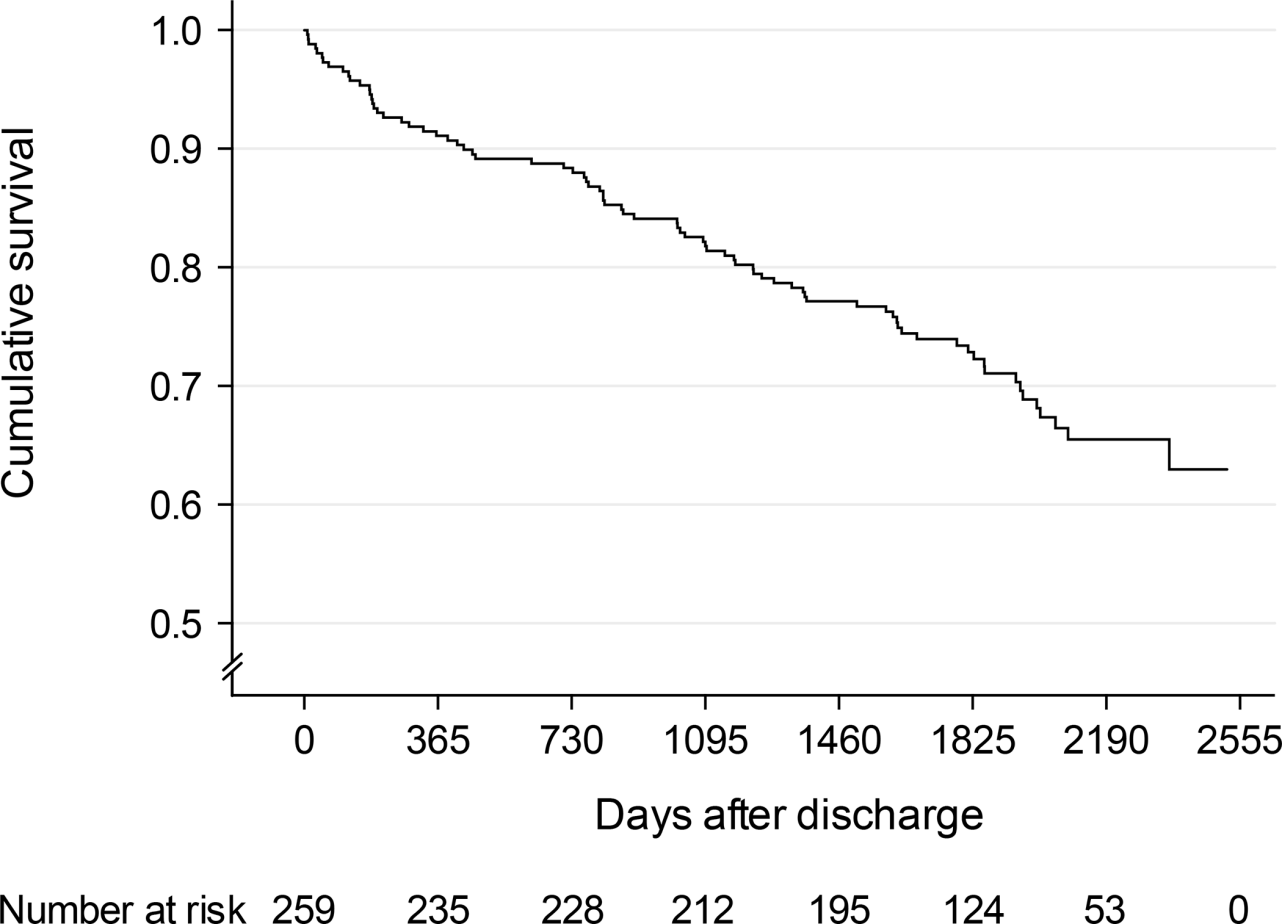
Kaplan-Meier plot of long-term survival for 259 patients discharged from hospital after treatment of community-acquired pneumonia. One patient was lost to follow-up (censored) at day 1.

After discharge, 79 (30.5%) of the 259 hospital survivors of CAP died during a median follow-up of 1804 days (range 1–2520 days). One patient was lost to follow-up (censored) at day 1. Cumulative 30-day, 1-, 3-, and 5-year survival rates were 98.8% (95% CI 97.4–100.0%), 91.1% (95% CI 87.6–94.6%), 82.2% (95% CI 77.5–86.9%), and 72.9% (95% CI 67.4–78.4%), respectively (Fig 1).

The expected number of deaths in the male population based on national rate data was 16.89. There were 49 observed deaths for male patients. This resulted in a SMR in our male cohort of 2.90 (95% CI 2.09–3.71; *P* < .001), as compared with the risk of mortality in the total male Norwegian population controlling for age distribution. Similarly, there were 30 observed deaths for female patients and the expected number of deaths was 14.63. Thus, the SMR in our female cohort was 2.05 (95% CI 1.32–2.79; *P* < .001).

### Causes of death

**Fig 2:**
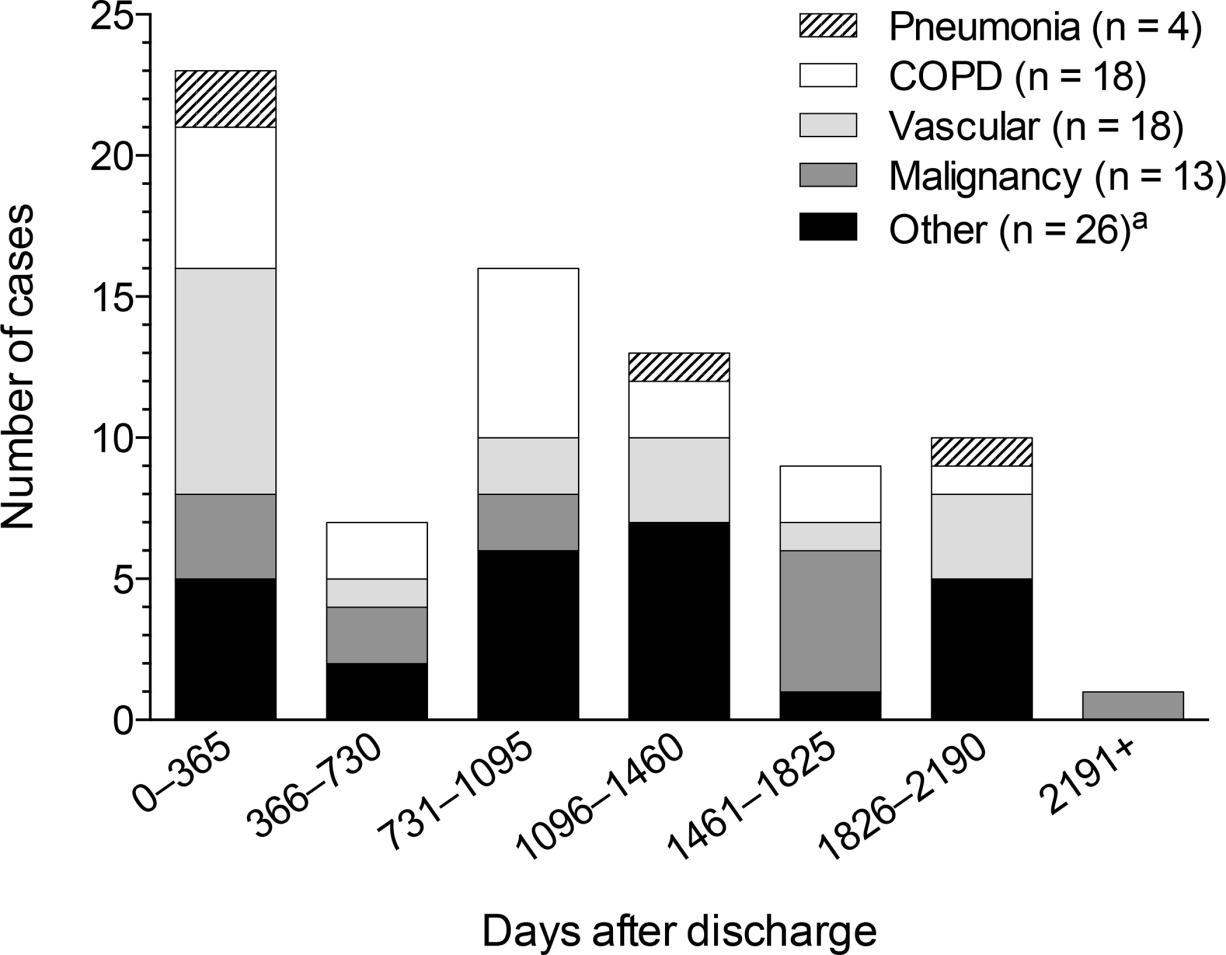
Causes of death by 1-year intervals following hospitalization for an episode community-acquired pneumonia. Abbreviations: COPD, chronic obstructive pulmonary disease. ^a^ Polyarteritis with lung involvement (Churg-Strauss), sepsis, unspecified infectious disease, urinary tract infection, hypoglycemia, diabetes mellitus with renal complication, tubulo-interstitial nephritis, kidney failure, other non-thrombocytopenic purpura, alcohol dependence syndrome, esophageal ulcer, ileus, cholecystitis, fistula of vagina to large intestine, gastrointestinal hemorrhage, cerebral palsy, motor neuron disease (2 cases), instantaneous or unattended death (2 cases), dementia (3 cases), accident (3 cases).

The main underlying causes of death during the follow-up period among the 79 non-survivors were COPD (23%), vascular diseases (23%) and malignancy (16%), while only 5% (4 cases) died because of recurrent pneumonia. Fig 2 shows the causes of death by 1-year intervals of follow-up. Vascular disease as the cause of death was most frequent in the first year after discharge. The other conditions did not show an excess mortality in the first year after discharge from the hospital.

### Baseline characteristics in relation to long-term all-cause mortality after hospitalization for CAP

The characteristics of patients according to survival status at the end of the follow-up period are presented in Tables 1 and 2. Median age of the patients was 66 years, 51% were male, and 64% had at least 1 comorbid condition. Seventy-five (29%) patients had a pure bacterial infection, 38 (15%) a pure viral infection, and 49 (19%) a mixed viral-bacterial infection; the microbial findings in these 162 (63%) patients are detailed in S1 Table. By univariable analysis, non-survivors were older and male, more likely to reside in a nursing home, to be severely ill on admission (CURB-65 score ≥3), and to have comorbidities including CVD, COPD, being immunocompromized, having renal disease, neurological disease, dementia, and having been vaccinated against pneumococcus and influenza, but less likely to be active smokers (Table 1). By contrast, there were no significant differences observed either for laboratory characteristics or for microbial etiology between survivors and non-survivors (Table 2). Inclusion of duration of symptoms did not alter the significance levels of any of the etiological variables appreciably.

**Table 1:** Demographic and clinical characteristics in 259 patients hospitalized for CAP, stratified according to long-term mortality.

Variable	Total (n = 259)	Alive^a^ (n = 180)	Dead (n = 79)	*P*^b^
**Demographics**				
Age (years)	66 (52–77)	61 (47–70)	78 (70–88)	< .001
Male sex, n (%)	133 (51.4)	84 (46.7)	49 (62.0)	.03
Active smoker, n (%)	65 (25.2)	57 (31.7)	8 (10.3)	.001
Nursing home resident, n (%)	2 (0.8)	0 (0.0)	2 (2.5)	.004
**Duration of symptoms (days)**^c^	4 (3–7)	5 (3–7)	4 (2–6)	.23
**Comorbid conditions, n (%)**				
CVD^d^	70 (27.0)	29 (16.1)	41 (51.9)	< .001
COPD	60 (23.2)	28 (15.6)	32 (40.5)	< .001
Immunocompromized^e^	46 (17.8)	24 (13.3)	22 (27.8)	.002
Autoimmune disease^f^	34 (13.1)	23 (12.8)	11 (13.9)	.59
Diabetes mellitus	33 (12.7)	20 (11.1)	13 (16.5)	.32
Renal disease	30 (11.6)	11 (6.1)	19 (24.1)	< .001
Neurological disease^g^	16 (6.2)	6 (3.3)	10 (12.7)	.01
Dementia	14 (5.4)	2 (1.1)	12 (15.2)	< .001
Liver disease	4 (1.5)	4 (2.2)	0 (0.0)	.40
**Vaccination status, n (%)**				
Influenza vaccination (<1 year)	64 (33.2)	39 (27.7)	25 (48.1)	.01
Pneumococcal vaccination (<10 years)	24 (12.3)	12 (8.4)	12 (23.1)	.004
**Severity of illness, n (%)**				
CURB-65 ≥3^h^	91 (36.5)	42 (24.3)	49 (64.5)	< .001
ICU admission	41 (15.8)	26 (14.4)	15 (19.0)	.33

Note: Data are presented as No. (%) or median (25th–75th percentile). Abbreviations: CAP, community-acquired pneumonia; CVD, cardiovascular disease; COPD, chronic obstructive pulmonary disease; CURB-65, Confusion-Urea-Respiratory-Blood pressure-65 score; ICU, intensive care unit.

^a^ One case was lost to follow-up (censored) at day 1.

^b^ Comparison between patients who were alive or dead at the end of follow-up.

^c^ Days of clinical symptoms at admission.

^d^ Coronary heart disease, heart failure, cerebrovascular disease, peripheral artery disease, aneurysm.

^e^ Primary or acquired immunodeficiency, active malignancy, immunosuppressive drugs.

^f^ Rheumatoid arthritis, systemic lupus erythematosus, inflammatory bowel disease, autoimmune hepatitis, Sjogren’s disease, psoriasis.

^g^ Central nervous disease, neuromuscular disease.

^h^ Patients were classified according to the CURB-65 severity scoring system (score ≥3, high-risk group).

**Table 2:** Laboratory and microbiological characteristics in 259 patients hospitalized for CAP, stratified according to long-term mortality.

Variable	Total (n = 259)	Alive^a^ (n = 180)	Dead (n = 79)	*P*^b^
**Laboratory and radiographic findings**				
Bilateral infiltrate, n (%)	62 (23.9)	40 (22.2)	22 (27.8)	.33
Leucocyte count (×10^9^/L)	12.4 (9.3–16.6)	12.2 (9.2–17.0)	12.9 (9.8–16.0)	.52
CRP (mg/L)	219 (104)	225 (104)	207 (103)	.29
Creatinine (μmol/L)	77 (63–97)	75 (62–92)	90 (66–107)	.05
Albumin (g/L)	28 (5)	28 (5)	27 (5)	.06
**Etiology of CAP, n (%)**^c^				
By category of agents				
Pure bacterial^d^	75 (29.0)	50 (27.8)	25 (31.6)	.58
Pure viral	38 (14.7)	26 (14.4)	12 (15.2)	.74
Viral–bacterial	49 (18.9)	37 (20.6)	12 (15.2)	.17
Unknown	97 (37.5)	67 (37.2)	30 (38.0)	.67
*Streptococcus pneumoniae*	80 (30.9)	59 (32.8)	21 (26.6)	.30
Influenza viruses	39 (15.1)	27 (15.0)	12 (15.4)	.63
Bacteraemia	24 (9.3)	19 (10.6)	5 (6.3)	.41

Note: Data are presented as No. (%), and mean (SD) or median (25th–75th percentile) depending on distribution. Abbreviations: CAP, community-acquired pneumonia; CRP, C-reactive protein.

^a^ One case was lost to follow-up (censored) at day 1.

^b^ Comparison between patients who were alive or dead at the end of follow-up.

^c^ Inclusion of duration of symptoms (in Table 1) did not change any of the results appreciably.

^d^ Reference group.

### Multivariable analysis

Table 3 shows the demographic, clinical, laboratory and etiological variables that were selected as candidates for the multivariable model based on their univariable association with long-term all-cause mortality (*P* < .15) in our cohort and the results from multivariable analysis. This final model based on the purposeful selection of variables method indicated that the risk of death increased by 83% per decade increase in the patients’ age. Patients with CVD, COPD and immunocompromization had a 2.63-fold, 2.09-fold, and 98% increased risk of death compared to those without these conditions, respectively. The model also indicated that the risk of death increased by 25% for each 5g/L decrease in the patients’ serum albumin level measured at hospital admission. Conversely, somewhat odd, active smokers had a 68% reduced risk of death compared to non-smokers; post hoc analysis revealed that active smokers were significantly younger than non-smokers (median age 57 vs. 68 years; *P* < .001). To avoid collinearity, renal disease and creatinin level were analyzed separately because they were strongly associated with each other. Importantly, according to the criteria, influenza and pneumococcal vaccination were not considered due to insufficient data. Also, nursing home resident was not entered into the model because there were too few occurrences (i.e., the contingency table contained a zero cell). The Cox-Snell residual plot indicated good fit of the model to the data (S1 Fig). The *c*-index was 0.84 indicating that the model had good discrimination.

**Table 3:** Multivariable analysis of factors associated with long-term mortality after hospitalization for CAP.

Variable	Univariable HR (95% CI)	Multivariable HR (95% CI)	*P*^a^
CVD^b^	3.92 (2.51–6.11)	2.63 (1.61–4.32)	< .001
COPD	2.68 (1.71–4.20)	2.09 (1.27–3.45)	.004
Immunocompromized^c^	2.15 (1.32–3.52)	1.98 (1.17–3.37)	.01
Age (years), per decade	1.96 (1.66–2.33)	1.83 (1.47–2.28)	< .001
Albumin (g/L), per SD	0.80 (0.64–1.01)	0.75 (0.58–0.96)	.02
Active smoker	0.28 (0.14–0.58)	0.32 (0.14–0.74)	.01
Nursing home resident^d^	7.85 (1.90–32.43)		
Dementia	5.48 (2.94–10.21)		
CURB-65 ≥3^e^	3.92 (2.45–6.28)		
Renal disease^f^	2.95 (1.76–4.95)		
Pneumococcal vaccination (<10 years)^g^	2.57 (1.35–4.91)		
Neurological disease^h^	2.48 (1.28–4.82)		
Influenza vaccination (<1 year)^g^	2.14 (1.24–3.69)		
Male sex	1.68 (1.07–2.65)		
Creatinine (μmol/L), per IQR^f^	1.14 (1.00–1.31)		

Note: Risk factors were assessed by Cox proportional hazards model using the purposeful selection of variables method. Of 259 cases, 7 had missing serum albumin values. The multivariable model was thus based on 252 subjects and the corresponding 74 failures. Abbreviations: CAP, community-acquired pneumonia; HR, hazard ratio; CI, confidence interval; CVD, cardiovascular disease; COPD, chronic obstructive pulmonary disease; SD, standard deviation; CURB-65, Confusion-Urea-Respiratory-Blood pressure-65 score; IQR, interquartile range.

^a^ Refers to multivariable analysis.

^b^ Coronary heart disease, heart failure, cerebrovascular disease, peripheral artery disease, aneurysm.

^c^ Primary or acquired immunodeficiency, active malignancy, immunosuppressive drugs.

^d^ Variable was not entered into multivariable model because there were too few occurrences.

^e^ Patients were classified according to the CURB-65 severity scoring system (score ≥3, high-risk group).

^f^ Creatinine and renal disease were analyzed separately because of their strong association (the same model was obtained).

^g^ Variable was not entered into multivariable model because data were insufficient.

^h^ Central nervous disease, neuromuscular disease.

## Discussion

Our results largely confirm substantial long-term mortality among hospital survivors of CAP and that chronic diseases, including COPD, vascular diseases and malignancy are major causes of death. Older age and the presence of CVD, COPD, immunocompromization, and low serum albumin level at hospital admission were identified as independent risk factors for long-term mortality, whereas active smoking was protective, probably due to the younger age of active smokers compared to non-smokers (*P* < .001). Results extend the prognostic value of albumin level at hospital admission to 5 years of follow-up after hospitalization for CAP. Importantly, pneumonia etiology had no prognostic value in these patients with etiologically well characterized CAP.

One- and 5-year mortality rates were 8.9% and 27.1% in our study. For inpatients with CAP, previous studies have reported 1-year mortality rates of 7–41% [8, 13, 36] and 5-year mortality rates of 36% to greater than 50% [2, 5, 37]. The variability in these mortality rates is probably best explained by differences in the studied populations [3, 4]. In the present study, mortality in males and females were 2.90-fold and 2.05-fold greater than expected compared with population data, respectively; both differences were statistically significant, falling within the range of 1.5–3.6 presented in other studies [6–9, 11]. However, these results must be interpreted with caution because, although controlling for age and sex, many factors that are well known to be important risk factors in patients with CAP were not controlled for (e.g., comorbidities). A 1.65-fold increase probably represents a fairly accurate estimate of overall increase in risk for long-term mortality associated with surviving an episode of CAP [9].

In our study, causes of death after CAP were mostly attributed to major chronic diseases and not to recurrent pneumonia, consistent with previous studies [7, 9–11, 14]. The incidence of vascular deaths was highest in the first year after CAP, whereas deaths from COPD and malignancy occurred at a more constant level during follow-up. By contrast, Bruns et al. [7] found that the incidence of deaths from malignancy was highest in the first year after CAP. However, our results provide further support that cardiovascular events may play an important role in long-term outcome of hospital survivors of CAP, especially in the first year after discharge [20, 22].

Consistent with previous studies, we found that older age and comorbid conditions, including CVD [11, 12, 22, 26], COPD [13, 16], and immunocompromization (i.e., malignancy, immunosuppressive conditions) [7, 11–13, 16] were independent risk factors for long-term mortality in patients with CAP. Our findings were contrary to a recent study [10] where diabetes was independently associated with long-term mortality. It is possible that our cohort of patients was less comprehensively defined with regard to diabetes or, alternatively, that comorbidities, in particular cardiovascular and renal diseases [38], were incompletely adjusted for in the multivariable models performed in the other study. Nevertheless, the study had some weaknesses, including small sample size and analysis was confined to patients with non-severe CAP, limiting the generalizability of the results.

Significantly, low serum albumin level at hospital admission was associated with long-term mortality in our study. This finding extends the results of previous studies that have identified hypoalbuminemia (albumin range, 35–50 g/L) as a prognostic marker of 28–30-day [30, 39] and 2.5-year mortality [11] in patients with CAP to 5 years. Importantly, hypoalbuminemia has been associated with worse outcomes including increased complications and reduced short-term and longer-term survival in critically ill patients [40]. Similar findings have also been demonstrated in patients with CAP [41]. Perhaps most notably among these complications are cardiac events [20, 22] as supported by our study. However, it remains uncertain whether the effect of hypoalbuminemia on outcome is a causal relationship or whether it is rather a marker of serious disease [40].

With regard to severity of illness, studies have mainly focused on the Pneumonia Severity Index [1], a more complex prediction rule than the CURB-65 score for predicting 30-day mortality in patients with CAP, indicating that it may also be accurate for predicting long-term mortality in these patients [2, 7, 16, 18, 19, 42]. Conversely, studies evaluating the CURB-65 score have had conflicting results [17, 18]. Although we found that non-survivors were frequently those classified into a high-risk group (i.e., CURB-65 score ≥3), the CURB-65 score was not found to be an independent risk factor for 5-year mortality. This is maybe not surprising since the CURB-65 score largely reflects acute physiological derangements; it is less age driven than the Pneumonia Severity Index and does not contain comorbidity components, both proven to be important risk factors for long-term mortality, as supported by our study. Thus the CURB-65 score is a valuable tool for predicting short-term mortality in CAP patients but it may not be optimal for assessing long-term prognosis.

Some previous studies have examined etiological factors and long-term mortality in patients with CAP [6, 11, 13, 24, 26–28]. These studies have indicated an association between specific CAP pathogens such as *S*. *pneumoniae* [6, 24] and gram-negative bacilli [11] and an increased mortality risk, or have obtained negative results [13, 26, 28]. Key limitations have been highly selected samples (i.e., only veterans with pneumococcal pneumonia) [24], insufficient data [27], and application of detection methods confined to traditional microbiological techniques (i.e., bacterial cultures, urinary antigen assays, serology) [6, 11, 13, 26, 28]. A unique feature of the present study was the systematic use of a broad range of PCR-based methods in combination with traditional techniques that allowed an etiologic diagnosis of CAP in most cases (63%). It is thus interesting to note that no significant association was found between microbial etiology and 5-year mortality. This finding corroborates that reported by Adamuz et al. [13], examining 1-year mortality, and does not support the notion that CAP caused by pathogens other than pneumococci may serve as marker for underlying physiological weakening [43]. However, given the wide variety of settings and diagnostics used across these few studies, and the diversity of the results, further research is indeed warranted.

Strengths of our study include its prospective design and that survival status was ascertained in nearly 100% of the patients included in the study. The extensive microbiological work-up that allowed the etiologic diagnosis of CAP in a large proportion of cases also represents a strength. However, several limitations should be considered. First, because this study was performed at a single institution, this may limit generalizability. Second, as the study design did not include a control group, we compared overall survival in our cohort to an age- and sex-standardized population retrieved from national life table data. Thus we were not able to control for important factors that are well known to be risk factors for long-term mortality in patients with CAP (e.g., comorbidities). Third, some patients who met the inclusion criteria were not enrolled in the study and the complete microbial sampling could not be applied in every patient. These issues are inherent in all studies enrolling patients with suspected CAP and assessing diagnostic techniques. We have no reason to believe that the patients who were not included would be substantially different compared with the study group. Samples to be obtained at admission were decided by the trained physicians on call and were completed on the ward according to the procedure by members of the project team within 48 hours of admission. Selection bias in the collection of samples is therefore possible but unlikely. Moreover, all patients were broadly categorized based on viral, bacterial, viral-bacterial infection, or no etiology. The heterogeneity of various bacterial and viral CAP pathogens within these groups may have restricted the ability to differentiate these potential risk factors. Fourth, this study did not evaluate alcohol abuse that potentially could have influenced disease severity, particularly in patients with pneumococcal CAP [44]. Also, we were not able to identify the impact of either vaccination status due to insufficient data, or of nursing home resident status because there were too few occurrences. Furthermore, we did not collect information on prior smoking history which probably would have had an influence on the somewhat disturbing finding that smoking turned out to be a protective factor against death in our study. Fifth, we used death certificates to determine causes of death and the accuracy of this approach has been questioned [45]. Nevertheless, a recent study found strong agreement between the death certificates’ stated causes of death and the causes of death determined by case studies conducted by physicians [7].

## Conclusions

The results of the present study largely confirm substantial comorbidity-related long-term mortality after hospitalization for CAP as well as the impact of several well characterized risk factors for death among these patients. Our findings extend the prognostic value of serum albumin level at hospital admission in patients with CAP to 5-year survival after discharge. Pneumonia etiology had no prognostic value, but this remains to be substantiated by further studies using extensive diagnostic microbiological methods in the identification of causative agents of CAP.

## Supporting Information

S1 Dataset Minimal data set underlying the findings in the study. Variable instructions are in sheet 2.
(XLSX)

S1 Fig Plot of the Cox-Snell residuals from the model. The curve is very close to the diagonal indicating a good model fit.
(EPS)

S1 Table Microbial findings in 162 hospital survivors with an etiologically established diagnosis of community-acquired pneumonia. (DOCX)

S1 Text The inclusion process for the study population. (DOCX)
