# Supplementary material for: Risk Factors for Long-Term Mortality after Hospitalization for Community-Acquired Pneumonia: A 5-Year Prospective Follow-Up Study
Source: PLoS One. 2016 Feb 5;11(2):e0148741. doi: 10.1371/journal.pone.0148741 (PMC4746118; doi:10.1371/journal.pone.0148741)
Supplement: S1 Table — (DOCX) [file pone.0148741.s003.docx]

**S1 Table. Microbial findings in 162 hospital survivors with an etiologically established diagnosis of community-acquired pneumonia.**

| **Pure bacterial and pure viral infections** | **n (%)** | **Viral–bacterial coinfections** | **n (%)** |
| --- | --- | --- | --- |
| **Pure bacterial (single)** | **60 (37)** | **Viral–bacterial (dual)** | **34 (21)** |
| *S. pneumoniae* | 37 (23) | *S. pneumoniae* plus: |  |
| *M. pneumoniae* | 7 (4) | Influenza viruses | 12 (7) |
| *C. pneumoniae* | 5 (3) | Rhinovirus | 8 (5) |
| *H. influenzae* | 2 (1) | Metapneumovirus | 2 (1) |
| *M. catarrhalis* | 2 (1) | Parainfluenza viruses | 1 (0.6) |
| *L. pneumophila* | 2 (1) | Respiratory syncytial virus | 1 (0.6) |
| *B. pertussis* | 2 (1) | *H. influenzae* plus: |  |
| Group A streptococcus | 1 (0.6) | Parainfluenza viruses | 2 (1) |
| *H. parainfluenzae* | 1 (0.6) | Influenza viruses | 1 (0.6) |
| *Enterobacteriaceae* | 1 (0.6) | Metapneumovirus | 1 (0.6) |
| **Pure bacterial (dual)** | **15 (9)** | Rhinovirus | 1 (0.6) |
| *S. pneumoniae* plus: |  | *B. pertussis* plus: |  |
| *H. influenzae* | 4 (2) | Influenza viruses | 3 (2) |
| *Enterobacteriaceae* | 1 (0.6) | Respiratory syncytial virus | 1 (0.6) |
| *L. pneumophila* | 1 (0.6) | *M. catarrhalis* plus: |  |
| *M. pneumoniae* | 1 (0.6) | Influenza viruses | 1 (0.6) |
| *B. pertussis* | 1 (0.6) | **Viral–bacterial (multiple)** | **15 (9)** |
| *L. pneumophila* plus: |  | *S. pneumoniae* plus: |  |
| *B. pertussis* | 2 (1) | *M. catarrhalis* and rhinovirus | 2 (1) |
| *Enterobacteriaceae* plus: |  | *H. influenzae* and enterovirus | 1 (0.6) |
| *M. pneumoniae* | 1 (0.6) | Group A streptococcus and influenza viruses | 1 (0.6) |
| *C. pneumoniae* | 1 (0.6) | *B. pertussis* and influenza viruses | 1 (0.6) |
| *H. influenzae* plus: |  | *B. pertussis* and rhinovirus | 1 (0.6) |
| *H. parainfluenzae* | 1 (0.6) | *C. pneumoniae* and parainfluenza viruses | 1 (0.6) |
| *M. pneumoniae* plus: |  | Influenza viruses and rhinovirus | 1 (0.6) |
| *B. pertussis* | 1 (0.6) | Parainfluenza viruses and adenovirus | 1 (0.6) |
| *D. pneumosintes* plus: |  | Metapneumovirus and rhinovirus | 1 (0.6) |
| *Prevotella* spp. | 1 (0.6) | Rhinovirus and enterovirus | 1 (0.6) |
| **Pure viral (single)** | **35 (22)** | *L. pneumophila* plus: |  |
| Influenza viruses^a^ | 15 (9) | *B. pertussis* and influenza viruses | 1 (0.6) |
| Rhinovirus | 11 (7) | *B. pertussis* and rhinovirus | 1 (0.6) |
| Metapneumovirus | 2 (1) | *H. influenzae* plus: |  |
| Parainfluenza viruses | 3 (2) | *B. pertussis* and rhinovirus | 1 (0.6) |
| Respiratory syncytial virus | 2 (1) | *Enterobacteriaceae* plus: |  |
| Enterovirus | 2 (1) | Influenza viruses and enterovirus | 1 (0.6) |
| **Pure viral (dual)** | **3 (2)** | … | … |
| Influenza viruses *plus:* |  | … | … |
| Rhinovirus | 1 (0.6) | … | … |
| Respiratory syncytial virus | 1 (0.6) | … | … |
| Rhinovirus *plus:* |  | … | … |
| Respiratory syncytial virus | 1 (0.6) | … | … |

Note: *S. pneumoniae, Streptococcus pneumoniae; M. pneumoniae, Mycoplasma pneumoniae; C. pneumoniae, Chlamydophila pneumoniae; H. influenzae, Haemophilus influenzae; M. catarrhalis, Moraxella catarrhalis; L. pneumophila, Legionella pneumophila; B. pertussis, Bordetella pertussis; H. parainfluenzae, Haemophilus parainfluenzae; D. pneumosintes, Dialister pneumosintes.*^a^ One patient was also coinfected with *Pneumocystis jirovecii*.
