# Supplementary material for: Risk Factors for Long-Term Mortality after Hospitalization for Community-Acquired Pneumonia: A 5-Year Prospective Follow-Up Study
Source: PLoS One. 2016 Feb 5;11(2):e0148741. doi: 10.1371/journal.pone.0148741 (PMC4746118; doi:10.1371/journal.pone.0148741)
Supplement: S1 Text — (DOCX) [file pone.0148741.s004.docx]

**S1 Text. The inclusion process for the study population.**

Adult patients (aged ≥18 years) with suspected pneumonia admitted to Medical Department, Drammen Hospital, Vestre Viken Health Trust in Norway were consecutively recruited between January 2008 and January 2011. A total of 320 patients were screened within the first 48 hours of admission. Of these, 33 (10%) patients were excluded (based on the predefined criteria) for the following reasons: previous hospitalization within the past 2 weeks (2 patients), chest radiograph was not performed (1), or no new infiltrate was detected (19), or noninfectious cause of lung infiltration and/or bronchial obstruction was revealed (7), or fever was not documented (4). A total of 287 patients (90% of the screened population) were eligible. Of these, 4 (1%) patients did not consent to enter the study. Sixteen patients who entered the study were subsequently withdrawn (6%, 16 of 287 patients) for the following reasons: consent withdrawal (1 patient), previous participation (2), reduced cooperation (2), missing or incorrect ID on case record form (3), inadequate sampling (2), or initial positive chest radiographic findings failed by review of radiologist (6). Of the remaining 267 patients who were included in the study and followed up, 8 (3%) died in the hospital.
